# Losses of natural coastal wetlands by land conversion and ecological degradation in the urbanizing Chinese coast

**DOI:** 10.1038/s41598-018-33406-x

**Published:** 2018-10-09

**Authors:** Qiaoying Lin, Shen Yu

**Affiliations:** 1 CAS Key Laboratory of Urban Environment and Health, Institute of Urban Environment, Chinese Academy of Sciences, Xiamen, 361021 China; 2 University of Chinese Academy of Sciences, Beijing, 100006 China; 3 Quanzhou Normal University, Quanzhou, 362000 China

## Abstract

Coastal wetland ecosystems have experienced serious losses of area and ecological function and are currently facing worldwide challenges due to coastal development and global climate change. This study attempted to explore patterns and possible factors driving loss of natural coastal wetlands due to land conversion (permanent loss) and ecological degradation (temporal loss) in three urbanizing coastal city clusters, China in the period of 1990–2015. The natural coastal wetland area was substantially lost due to land conversion highly related to regional economic development. The ecological degradation, assessed as a function of surface water quality, resulted in much greater impairment area of natural coastal wetlands. This impairment was predominantly driven by inbound river pollutants’ discharge, rather than local discharge. This study suggests that the ecological degradation should be considered as well as the land conversion loss for conserving the remaining natural coastal wetland ecosystems. The pollutant discharges from the inbound river watersheds need to be mitigated as the local discharges for reducing the functional degradation of the natural coastal wetlands while the regional economic development plan should consider the conservation needs of the remaining natural coastal wetlands worldwide.

## Introduction

Coastal wetland ecosystems, an ecotone between terrestrial and marine ecosystems, are vulnerable to current global changes, including sea level rise^1,2^, extreme storms^3^, and human impacts^2,4–6^. Due to rapid economic development during the 20^th^ century, annual global losses of 0.7–1.2% have cumulatively resulted in approximately 63% of all coastal wetland losses^7^. Estimated total coastal wetland loss due to human activities is greater than 67% of its original area, including 65% of sea grass and 48% of other submerged aquatic vegetation^5^. About 25–50% of the world’s coastal wetlands reclaimed were converted to agricultural lands during the 20^th^ century^2,8^. In addition to the direct land or habitat loss, 762 global coastal wetland sites were found to be eutrophic and/or hypoxic^9^. Eutrophication is considered one of the main causes of salt marsh functional degradation^10,11^ or loss^12^.

Coastal zones are among the most urbanized regions in the world and approximately 37–40% of the world’s population lives within 100 km of the coastline^13,14^. As hot spots of global development, coastal countries and regions simultaneously become the hot spots of land loss and ecological degradation of coastal wetlands^7^. Due to coastal wetland loss, twelve of the most diverse and productive estuaries and coastal seas have lost 99.0% of formerly important species and 96.5% of seagrass and wetland habitats have been destroyed^5^. Simultaneously, global rivers discharged 43.2 Tg N, 8.6 Tg P, 304 Tg C, and 14,500 Tg total suspended solids (TSS) to their coasts in 2000^15^.

Intensive human activities, such as urbanization, have been identified as key causes of land loss and ecological degradation of coastal wetlands^2,16^. United States annually lost 324 km^2^ of coastal wetlands between 2004–2009^17^ and Australia lost or converted nearly 30% of its coastal wetland between 1997–2002^18^. Spain lost 45% of ecosystem services of coastal wetlands by 2013 due to ecological degradation and unsustainable utilization^19^. On the other hand, the developing countries in Africa and Asia are experiencing much faster urbanization than other regions^20^. Asia lost coastal wetlands at an annual rate of 1.1% of the total area, which is greater than Europe (annually 0.99% of the total area) or North America (annually 0.51% of the total area)^7^. The Mekong River Delta in Vietnam lost 1,240 km^2^ of mangrove wetlands between 1954–1974^21^. The Yellow Sea region in East Asia lost 28% of tidal flats since 1980s with an annual rate of 1.2% of the total area^22^.

China has experienced the fastest economic development in the world. With the rapid urbanization in the last 40 years, 43% of China’s population was living in the coastal provinces by 2015^23^. Accordingly, 16,878 km^2^ of coastal wetlands were reclaimed from 1949 to 2008, of which over 11,163 km^2^ were reclaimed between 1979–2014^24^, and more than 5,880 km^2^ are planned for reclamation by 2020^25^. In total, China has lost about 53% of its temperate coastal wetlands, 73% of its mangrove marshes, and 80% of its coral reefs in the last 50 years^26^. Furthermore, 86% of existing estuaries, bays, coral reefs, and other marine ecosystems in China were in an ecologically sub-healthy/unhealthy state, and 80% of existing estuaries and 57% of bays were eutrophic by 2015^27^. This environmental deterioration is accompanied by significant biodiversity declines in coastal wetlands. For instance, seaweed density dropped from 278 plants m^−2^ (2011) to 181 plants m^−2^ (2015) in Beihai, Guangxi Province; species of reef-building corals decreased from 52 (2011) to 36 (2015) on the east coast of Hainan Province^27^.

Coastal wetlands are facing two types of stress under development pressures, i.e. land conversion loss and ecological degradation. Conversion of coastal wetlands to other land uses (including agricultural and urban lands) is a major contributor to coastal wetland loss^2^, readily observable by local administrations and the public. However, wetland ecological degradation is only an academic concern, often ignored by the government and the public. While some ecologically-degraded wetlands might be resilient if supported by sound protection measures, most land conversion loss is irreversible^5,28,29^. Therefore, in order to protect wetland resources and identify possible driving mechanisms of wetland loss, it is important to discriminate between the contributions to coastal wetland loss as a result land conversion versus ecological degradations. This study hypothesizes that coastal wetland loss (temporal or permanent) due to ecological degradation (such as eutrophication and hypoxia) might be crucial for the survival of coastal wetlands remaining after land conversion losses. Pollution, from both local discharges and inbound river contamination, could be an important driver of ecological degradation.

**Figure 1:**
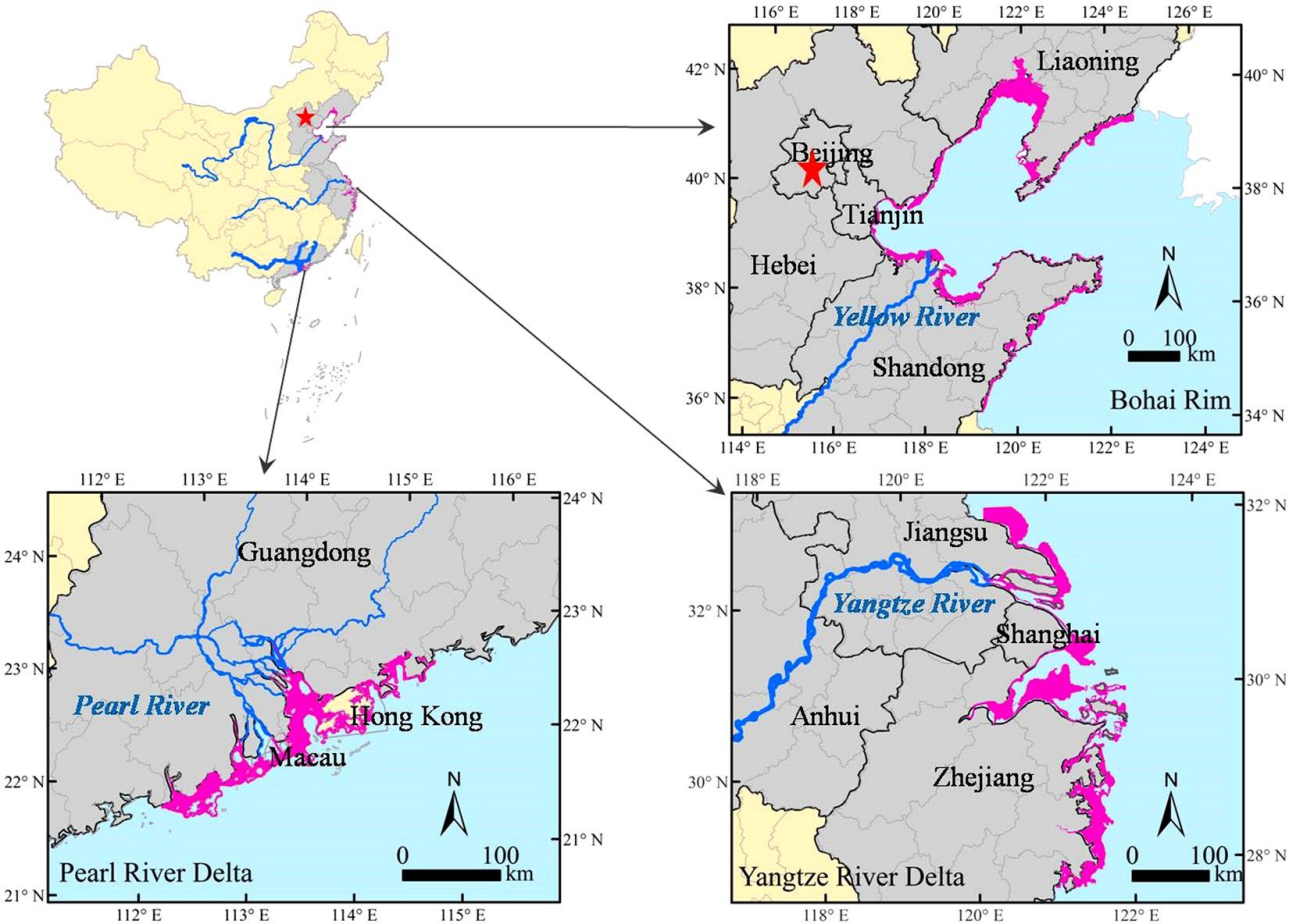
Locations of three developed city clusters along the Chinese coastline, the remaining natural coastal wetland resources (2015), and related trans-provincial inbound rivers. Data were interpreted from images of LandSat and the Global terrain model (Table S2) obtained at the Geospatial Data Cloud site, Computer Network Information Center, Chinese Academy of Sciences (http://www.gscloud.cn/) and NOAA National Geophysical Data Center (NGDC) of USA (http://www.ngdc.noaa.gov/mgg/global/global.html) ^49^.

Three Chinese coastal city-clusters (Fig. 1), Bohai Rim (BHR), Yangtze River Delta (YRD), and Pearl River Delta (PRD), have formed since the 1980s. These clusters are located in estuaries of the three biggest rivers in China, the Yellow, Yangtze, and Pearl Rivers. These estuaries contained 36.4% of the national population, contributed 50.8% of the 2015 national gross domestic production (GDP) (Table S1), and received approximately 75% of the national river pollutant discharges in 2015^27^. This study analyzed these cluster regions to explore temporal and spatial patterns of coastal wetland losses via land conversion and ecological function degradation during the rapid development period of 1990–2015. The three city clusters represent different Chinese economic modes, which might provide an understanding of possible driving mechanisms of coastal wetland losses in relation to regional economic development and pollution. This study would be beneficial for coastal wetland resource protection in other countries experiencing rapid coastal development.

## Results

### Loss pattern of natural coastal wetland resources

**Figure 2:**
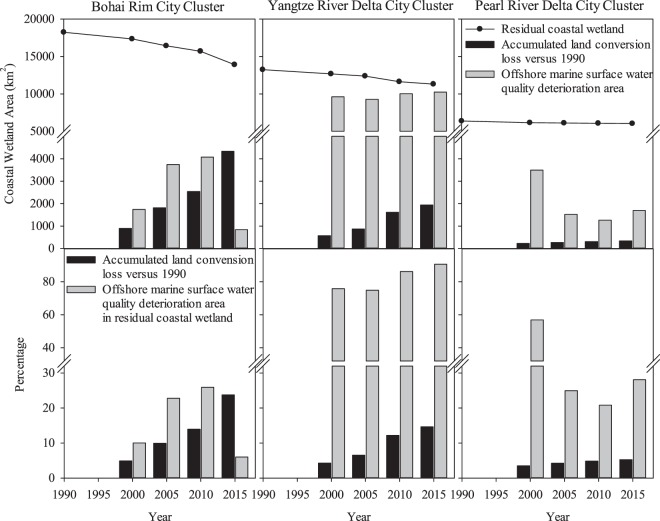
Natural coastal wetland changes in area via land conversion and ecological degradation in the three city clusters, China over time from 1990 to 2015. Upper charts present accumulated land conversion loss in area versus 1990 and offshore marine surface water quality deterioration in area of the investigation year, and lower charts are percentages of accumulated land conversion loss versus 1990 and offshore marine surface water quality deterioration in area of the remaining coastal wetland by the investigation year.

In comparison to the natural wetland area in 1990 (reference year), coastal wetlands in the regions of the three city clusters shrank in area (Fig. 2). The BHR region had the largest irreversible wetland conversion loss. Approximately 23.7% of the 1990 natural coastal wetland area of 18,243 km^2^ disappeared by 2015 (13,912 km^2^ remained). Land conversion losses of natural coastal wetlands in the YRD and PRD regions were about 14.7% (1,941 km^2^) and 5.3% (335 km^2^) of their 1990 areas (YRD: 13,248 km^2^ and PRD: 6,373 km^2^) by 2015, respectively (Fig. 2).

The ecological degradation of remaining natural coastal wetlands was defined by surface water quality, which represents a consequence of multiple ecological degradations according to the literature. Deterioration of water quality was defined as lower than Class III of the national standard (GB3097-1997), Table S5). In this study, completely different water quality patterns were observed between the three city cluster regions (Fig. 2). Areas of ecological degradation in the remaining natural coastal wetlands were extracted from the National Environmental Pollution Bulletins of China in years 2000, 2005, 2010, and 2015. The ecologically degraded area was significantly greater than the accumulated land conversion losses in the investigated regions except in BHR region in 2015 (Fig. 2). In the BHR region, the yearly ecologically-degraded area of the remaining natural coastal wetlands increased from 2000 (1,742 km^2^, 10.0% of remaining wetland area) to 2010 (4,073 km^2^, 25.9% of the remaining wetland area) but dropped in 2015 down to 838 km^2^, 6.0% of the remaining wetland area. However, the PRD region was reversed, i.e. the yearly ecologically-degraded area of the remaining natural coastal wetlands declined from 2000 (3,495 km^2^, 56.8% of the remaining wetland area) to 2010 (1,262 km^2^, 20.8% of the remaining wetland area) but increased in 2015 up to 1,695 km^2^, 28.1% of the remaining wetland area. The YRD region had the largest yearly ecologically-degraded area among the three city cluster regions, which covered 75.9% of the remaining wetland area (9,618 km^2^) in 2000 and increased up to 90.6% of the remaining wetland area (10,245 km^2^) in 2015.

Results of ANOVA-Repeated Measures indicated that both the investigated period and location significantly differentiated the land conversion loss of the natural coastal wetlands in the three city cluster regions. However, the area of yearly ecological degradation was only significant with the investigated period (P < 0.05, Table 1). The interactive effects of time and location were only significant for the land conversion loss (P < 0.01, Table 1).

**Table 1:** Effects of period and location on accumulated land conversion and yearly ecological degradation of the natural coastal wetlands in area in the three city cluster regions explored by analysis of variance (ANOVA)-Repeated Measures.

Item	df	Land conversion loss				Yearly ecological degradation			
		Accumulated loss area, km^2^ since 1990		% of coastal wetland area in 1990		Area, km^2^ in the investigation year		% of remaining coastal wetland area in the investigation year	
		F	P	F	P	F	P	F	P
Period	1	77.9	<0.001	100.5	<0.001	7.6	0.022	8.1	0.019
Location	2	20.1	<0.001	12.4	0.003	3.2	0.090	3.1	0.097
Interaction	2	20.3	<0.001	12.5	0.003	3.2	0.087	3.1	0.094

### Land conversion pattern of the natural coastal wetland loss

Annual land conversion loss of natural coastal wetlands shifted over the four investigated periods (1990–2000, 2000–2005, 2005–2010, and 2010–2015). The BHR region had increasing annual land conversion loss from 89.5 km^2^ yr^−1^ (1990–2000) up to 358 km^2^ yr^−1^ (2010–2015), while the PRD region had a reverse trend that decreased wetland land loss from 22.5 km^2^ yr^−1^ (1990–2000) to 5.4 km^2^ yr^−1^ (2010–2015) (Table 2). Land conversion loss in the YRD region peaked at 150 km^2^ yr^−1^ during 2005–2010; losses in the other three periods ranged from 56.8 to 65.2 km^2^ yr^−1^ (Table 2).

**Table 2:** Annual loss of the natural coastal wetlands and portions of new urban land conversions from the natural coastal wetland and converted agricultural land in the three coastal city cluster regions, China.

Investigation interval	Annual loss of the natural coastal wetland (km^2^ yr^−1^)			New urban land conversion					
				% of the natural coastal wetland loss			% of new urban land from the converted agricultural land use		
	BHR	YRD	PRD	BHR	YRD	PRD	BHR	YRD	PRD
1990–2000	89.5	56.8	22.5	1.6	1.2	16.6	0.0	0.0	0.0
2000–2005	183.5	59.3	9.1	5.2	1.8	33.2	10.8	29.9	54.2
2005–2010	145.8	150.1	7.6	3.9	0.2	42.6	21.4	67.2	65.5
2010–2015	357.9	65.2	5.4	16.9	6.1	82.8	27.8	85.5	10.4

**Figure 3:**
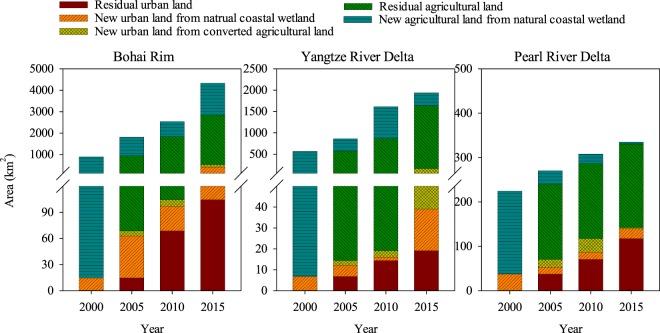
Conversions of coastal wetlands to agricultural and urban lands in the period of 1990–2015. Coastal wetland area in 1990 was set as a baseline and wetland land losses were calculated from this baseline. Remaining agricultural and urban lands refer to the area of these land uses in the previous investigation year. New agricultural and urban lands refer to coastal wetland conversions from the previous investigation year. New urban land converted from agricultural land use represents lands that were converted from coastal wetland to agricultural land uses in previous investigation years.

Natural coastal wetlands underwent a major conversion to agricultural land uses in the BHR and YRD regions during all four periods investigated (Fig. 3). Natural coastal wetland conversion to urban land use was a small, but gradually increasing proportion, nearly 16.9% and 6.1% of wetland conversion loss in the period of 2010–2015 for the BHR and YRD regions, respectively (Table 2). The PRD region exhibited a different wetland conversion pattern, especially in the period of 2010–2015, when 82.8% of wetland loss was directly due to conversion to urban land use (Fig. 3 and Table 2). Conversion to urban land use from wetlands previously converted to agricultural land uses contributed 28% and 86% of the urban land area in the BHR and YRD regions, respectively (Table 2). The PRD region increased its portion of urban land from converted agricultural land use to 66% in the period of 2005–2010, which dropped to 10% in the period of 2010–2015 (Table 2).

### Relationships of land conversion and ecological degradation of the natural coastal wetlands to economic development and pollutant discharges

Annual loss of natural coastal wetlands was significantly and positively correlated with annual new agricultural land conversion and regional agricultural GDP (P < 0.01, Table 3). These two factors were significantly and positively correlated interactively as well (P < 0.01, Table 3). Annual new urban land from natural coastal wetland and converted agricultural lands had significant and positive correlations with annual loss of the natural coastal wetlands (P < 0.01), as well as annual new agricultural land conversion (P < 0.05, Table 3). Annual new urban land conversions were strongly related to the regional GDP and its components (P < 0.01), but not significantly related to the GDP per capita (Table 3).

**Table 3:** Coefficients of Pearson correlations among land conversion loss of the natural coastal wetland loss and regional gross domestic product (GDP) and its components in four investigation periods and the three city cluster regions (n = 12).

Item	Annual new agricultural land	Annual new urban land	Regional GDP	Regional agricultural GDP	Regional industrial GDP	Regional service GDP	GDP per capita
Annual loss of natural coastal wetland	0.994**	0.770**	0.552	0.763**	0.545	0.396	0.158
Annual new agricultural land from natural coastal wetland		0.705*	0.523	0.730**	0.484	0.350	0.121
Annual new urban land from natural coastal wetland and converted agricultural land			0.745**	0.823**	0.762**	0.733**	0.505

**Represents a significant difference at P < 0.01 and *at P < 0.05.

The yearly ecological degradation area of the remaining natural coastal wetlands was significantly associated with annual discharges of chemical oxygen demand (COD_w_), which positively contributed 85% of the model R^2^, P < 0.01) and annual discharges of NH_4_^+^-N_w_ (negatively15% of the model R^2^, P < 0.05) from the main inbound river watersheds. These factors were determined by stepwise regression analysis among the discharges of COD, NH_4_^+^-N, total P, and oils from both the main inbound river watersheds and coastal regions (P < 0.01, n = 24, Table 4). In pollutant discharges from the main inbound river watersheds alone, COD_w_ and Oil_w_ positively contributed 70.7% (P < 0.01) and 7.6% (P < 0.05) of the model R^2^, respectively, and NH_4_^+^-N_w_ negatively contributed 21.7% of the model R^2^ (P < 0.05) (the model P < 0.01, n = 12, Table 4). The pollutant discharges from the investigated regions were also significantly associated with the yearly ecological degradation area of the remaining natural coastal wetlands. However, the significance level (P < 0.05, n = 12) was weaker and COD_r_ was the only significant variable (Table 4). By using GDP or its components (except the agricultural GDP) as a weighting factor, the performance of the linear models remarkably improved, increasing the model significance level to P < 0.01 (n = 12); under this scenario, COD_r_ (positive) and NH_4_^+^-N_r_ (negative) were two major variables (Table 4). The weighting of the service GDP gained the best improvement of the linear regression model (P < 0.01, R^2^ = 0.83) and the weighting of the GDP per capita also substantially improved the model (P < 0.01, R^2^ = 0.80, Table 4).

**Table 4:** Yearly ecological degradation area of the remaining natural coastal wetlands in the three city clusters of China in response to pollutant discharges from the coastal regions and main inbound river watersheds (Stepwise Regression, variables with partial P < 0.5 staying in the model when the model P, alpha < 0.05).

Discharge source		F	P	R^2^	Variable	Estimated parameter	Semi-partial R^2^	Partial P
Main inbound river watersheds and coastal regions (n = 24)		29.56	0.002	0.92	COD_w_^†^	0.002	0.78	0.004
					NH_4_^+^-N_w_	−0.030	0.14	0.029
					Intercept	1257		
Main inbound river watershed (n = 12)		26.78	0.001	0.92	COD_w_	0.002	0.65	0.003
					Oil_w_	0.054	0.07	0.043
					NH_4_^+^-N_w_	−0.037	0.20	0.011
					Intercept	695		
Coastal regions (n = 12)		6.95	0.034	0.50	COD_r_	0.066	0.50	0.034
					Intercept	−249		
Weighted by	GDP	10.40	0.011	0.78	COD_r_	0.158	0.51	0.030
					NH_4_^+^-N_r_	−0.799	0.26	0.037
					Intercept	−703		
	Industrial GDP	11.31	0.009	0.79	COD_r_	0.152	0.47	0.042
					NH_4_^+^-N_r_	−0.814	0.32	0.023
					Intercept	−473		
	Service GDP	14.94	0.005	0.83	COD_r_	0.153	0.49	0.036
					NH_4_^+^-N_r_	−0.839	0.34	0.013
					Intercept	−449		
	GDP per capita	12.04	0.008	0.80	COD_r_	0.155	0.56	0.021
					NH_4_^+^-N_r_	−0.774	0.24	0.036
					Intercept	−520		

^†^COD, NH_4_^+^-N, TP, and oils discharged from the main watersheds or coastal regions are labeled with a footnote of “w” or “r”, respectively.

## Discussion

Land conversion loss of the natural coastal wetlands was substantial in the three city cluster regions. This wetland loss was significantly correlated with converted agricultural and urban land uses. Numerous studies have pointed out that the land conversion loss of coastal wetlands directly reduces habitats of fish, birds, and other wildlife and threatens ecosystem services, including water filtration, biodiversity support, and flood abatement^5,30,31^. In this study, the yearly ecological degradation area of the remaining natural coastal wetlands were comparable (the BHR region) to or much larger (the YRD and PRD regions) than the accumulated land conversion loss of the natural coastal wetlands in the three city cluster regions between 1990–2015 (Fig. 2). Especially, the YRD city cluster region had 75%–91% of the remaining natural coastal wetlands (approximately 10,000 km^2^) between 2000 and 2015 (Fig. 2).

The natural coastal wetlands in the three city cluster regions received increased amounts of nutrient inputs in the years investigated. Coastal aquatic pollution causes eutrophication and/or hypoxia, which alters macro- and micro-algal, seagrasses, and aquatic animal communities and their functions in the coastal wetland ecosystems^16,32–34^. Eutrophication and hypoxia have been identified as major causes of salt marsh ecological degradation^12,35,36^. These changes lead to serious ecological destruction, economic loss, and public health risks^32–35^. Many failures of coastal wetland restorations suggest that the ecological degradation of the natural coastal wetlands is mostly irreversible^5,29,31^.

However, studies of ecological degradation of the natural coastal wetlands has mainly considered impacts from local or coastal regions^12,37,38^. This study indicated that discharges of nutrients and pollutants from trans-provincial inbound river watersheds were greater than direct discharges from the three coastal city cluster regions (Table S4). The Chinese coast receives 18 to 258 times the nutrient and pollutant discharges from major river watersheds (most trans-provincial rivers), compared with the coastal region discharges^27,39^. In 2015, the major river watersheds discharged into the Chinese coast 2.6 million tons of inorganic N and 0.26 million tons of total P, and 35 tide events covering an area of 2,809 km^2^ occurred^27^. In this study, the stepwise linear regression analysis confirmed that NH_4_^+^-N and COD discharges from the main inbound river watersheds, rather than those from the city cluster regions, were correlated with the yearly area of ecological degradation in the remaining natural coastal wetlands in the investigated city cluster regions (Table 4). These results suggest that mitigating the pollution of the coastal wetlands must address watershed discharges as well as controlling the local discharges.

The ecological degradation of remaining natural coastal wetlands is often attributed to anthropogenic land conversions, which lead to dramatic increases of energy, water, and fertilizer consumption^40^ that eventually impair ecosystem services provided by wetlands^14^. The largest land conversion of the natural coastal wetlands in the three city cluster regions was to agricultural land use (Fig. 2), suggesting that discharges of nutrients to the coastal wetland ecosystems might increase due to fertilization for crop production and excess feeding for high-density aquaculture.

Aquaculture, an expanding agricultural industry, leads to discharges of nutrients and pollutants (such as antibiotics) to the coastal wetland ecosystems^41,42^ and disturbs the coastal physical environment because of intensive cultivation activities^34,43^. For example, fish pond aquaculture alone discharged about 2,082 tons of nitrogen, 376 tons of phosphorus, and 5,056 tons of biological oxygen demand (BOD) to Yellow Sea and Bahai Sea in 2002^42^. In this study, an evident reduction of the ecological degradation area in the remaining natural coastal wetland of Bohai Rim in 2015 (Fig. 2) might be attributed to the policy lag effect of the Bohai Sea Environmental Protection General Planning (2008–2020) issued in 2009 by China^44^. Meanwhile, significant reductions of total N and P discharges in the Bohai Rim in 2010 and 2015 were found in comparison with their discharges in 2005 as well (Table S4).

Urbanization in watersheds increase river discharges of land derived sediment and pollutants to coastal wetlands^6,45,46^. Our results confirmed that urban land use was one of key land conversions of the natural coastal wetland in the three city cluster regions of China, especially in the latest decade, including direct land conversion from natural coastal wetlands and indirect conversion from the converted agricultural lands (Table 2 and Fig. 3). The urban land conversion rate changed over time and different at sites (Table 2), and as hypothesized, was related to regional development patterns.

Compared with natural factors, such as geological structure, sea level rise, and erosion, the economic development patterns were identified as an important factor in land conversion losses of world natural coastal wetlands^4,47^. Comparing coastal ecosystem changes before and after 1978 in China, He *et al*.^48^ also concluded that economic growth is directly associated with degradation of coastal ecosystems. In this study, the BHR region had the largest agricultural GDP among the three city cluster regions with considerable agricultural (including aquaculture) land conversion. The PRD region was dominated by a non-agricultural economy, with the greatest portion of urban land conversion among the three city cluster regions. The YRD region was also subject to non-agricultural economy, with large portion of new urban land conversion from the converted agricultural lands over time. Therefore, it is crucial to consider the local or regional economic development pattern and needs in order to assess and mitigate coastal wetland loss. However, Meng *et al*.^24^ found that the driving mechanism of land reclamation from sea in China was complicated. Weak relationships were found between economic indicators and statistical land reclamation area by the State Oceanic Administration of China (SOAC) in their study. Meaningfully, only marine industrial related indicators were positively related to the land reclamation area, such as marine industrial employees, proposition of marine industrial GDP in total GDP, added values of offshore oil and natural gas, and added values of marine chemicals, and mariculture area. In comparison with their study, this study used annual land conversional areas for urban and agricultural uses in the four investigated periods obtained from satellite-image interpretation instead of the SOAC-statistical reclamation area and used city-based economic data instead of the provincial data (except Shandong and Hebei provinces which were too big to represent^24^) (Table 3). On the other hand, the agricultural land use as explored in this study has been an important driving factor for land reclamation from sea to maintain the cultivated land requisition-compensation balance^24^.

### General limitations of this study

This study was carried out on the basis of available data. For example, the best resolution of ETOPO 1 (~10 m)^49^ was used to generate the seaward boundary of coastal wetlands according to the RAMSAR definition (<6 m). The offshore marine surface water quality area was extracted from the only available official national map of the Annual National Environmental Bulletin (Chinese Ministry of Environmental Protection) given that the annual monitoring data were not publicized. The pollutant discharges from the inbound river watersheds and coastal cities were only collected from the available years close to the investigation years (Table S2), i.e. 2001 for 2000 and 2006 for 2005 of coastal discharges and 2003 for 2000 of inbound river watersheds. On the other hand, the landward boundaries of natural coastal wetland in 1990 and land use conversion afterwards were defined using LandSat images instead of high resolution satellite images. Seawall, steep cliff, and boundary of aquacultural ponds were used as signs and images of 1989, 1990, and 1991, available images on different dates in 1990, and images of Google Earth were referenced. It is believed that tidal effects on coastal wetland boundaries would limit the interpretation accuracy.

Secondarily, ecological degradation was only defined by the offshore marine surface water quality deterioration based on yearly monitoring data mapping, which is the only available dataset at a national scale from 301 monitoring points. Accuracy of the dataset cannot be confirmed by this study. Other indicators, especially biological indicators, were not included in this study due to lack of availability. Future studies require comprehensive datasets to evaluation ecological degradation or ecosystem services.

In addition, although pollutant discharges from the rivers’ watersheds and coastal zones had been tested for correlations to offshore marine surface water quality deterioration, the impacts of land conversion, including physical disturbance, sedimentation, anthropogenic activities and others, were not identified in this study. Pollution incidents in the three coastal city cluster regions were not included in the study.

## Conclusions

This study compared losses of natural coastal wetlands in three city cluster regions (BHR, YRD, and PRD) in China between 1990–2015. Ecological degradation caused serious temporal loss of the remaining natural coastal wetlands in coincidence with the land conversion loss during the urbanizing period. The ecological degradation was associated with regional economic development (regional GDP and its components). Similar to most developing countries, the majority of natural coastal wetland conversion losses went to agricultural land use, including aquaculture in this study. Wetland conversions to urban land use was increasingly enhanced with regional economic development. Comparison of pollutant discharges from the coastal regions and the inbound rivers’ watersheds suggest that mitigation of ecological degradation or loss of natural coastal wetlands must consider reduction of pollution inputs with a land-sea integrative strategy in the future.

## Materials and Methods

### Study sites

The three city clusters, Bohai Rim (BHR), Yangtze River Delta (YRD), and Pearl River Delta (PRD), are located at the east coast from north to south and the most developed zones of China (Fig. 1). According to the statistical data of the year 2015^23^, the three investigated city clusters, involving 35 coastal cities of 8 provinces, host 36.4% of the national population and contribute 50.8% of the national GDP in 2015 (Table S1).

### Data source and processing

#### LandSat images and interpretation

LandSat images of the three coastal city clusters were interpreted for the coastal wetland distribution maps for1990, 2000, 2005, 2010, and 2015, obtained from the Global Land Cover Facility (GLCF) and Geospatial Data Cloud Site at the Computer Network Information Center, Chinese Academy of Sciences (Table S2). Twenty-three scenes of LandSat images cover the coastal wetland areas in the three city cluster regions for each investigated year, consisting of 15 scenes for the BHR, 4 scenes for the YRD, and 4 scenes for the PRD. The images for 1990, 2000, 2005, and 2010 were captured by LandSat 5 Thematic Mapper (TM) and LandSat 7 Enhanced Thematic Mapper Plus (ETM^+^) and the images for 2015 were by LandSat 8 Operational Land Imager (OLI) and all were at a 30-m spatial resolution. The cloud-free or less cloud (<1%) images were selected.

The downloaded LandSat images were preprocessed with color composite and registration. The LandSat 5 TM and LandSat 7 ETM^+^ images were composited with the bands of 5, 4, and 3 and the LandSat 8 OLI with the bands of 6, 5, and 4. The images for 2010 were used as the reference to register images for 1990, 2000, 2005, and 2015 using a second-order polynomial with a root mean square error (RMSE) smaller than half a pixel in both X and Y dimensions. All images are registered into the Lambert Conformal Conic coordinate system with the ERDAS Imagine (V9.2) platform software package.

The coastal wetland distribution in 1990 acts as the reference and its landward boundary was set as the initial boundary of the coastal wetlands for other investigation years. The landward boundaries of coastal wetland distribution in regions of the three city clusters in 1990 were defined using evident signs of seawall, steep cliff, and the boundary of aquacultural ponds and via comparisons among images of 1989–1991, images of available images on different dates in 1990, and images of Google Earth. The seaward boundary of coastal wetlands was defined according to the Ramsar Convention on Wetlands (<6-meter depth below the sea level) and extracted from the global terrain model^49^ (ETOPO 1, approximately 10-meter resolution, Table S2) with depth interpolation using the 3D analysis of an ArcGIS® 10 platform. Land use types of the converted coastal wetlands beyond this boundary seaward were visually interpreted and classified as three categories in the following year, i.e. natural coastal wetland, agriculture land, and urban land using the ArcGIS® 10 platform (Table S3). The images from Google Earth were also used as a reference for interpretation and validation of land use types. China’s city administrative maps were used to compile the boundaries of the three city cluster regions.

### Environmental and economic data collection

Offshore marine water quality and pollutant discharges from main inbound rivers and local coastal regions in the three city cluster regions were collected (Table S2). Raster maps of the offshore marine water quality were extracted from the National Environmental Bulletins of the Ministry of Environmental Protection of China for 2000, 2005, 2010, and 2015 to characterize surface water quality deterioration. According to the National Environmental Bulletins, the map of offshore marine surface quality was based on the annual monitoring data at the state monitoring points (301 points in 2015). The raster maps of marine water quality were registered with China’s city administrative map in the Lambert Conformal Conic coordinate system and digitally generate their vector maps using the ArcGIS® platform. The pollutant discharges (chemical oxygen demand (COD), NH_4_^+^-N, total phosphorus (TP), and oils) from watersheds of the Yellow River, the Yangtze River, and the Pearl River were record by the State Ocean Administration of China in 2003. The data for 2003 (substituting for 2000), 2005, 2010, and 2015 were collected while the pollutant discharges from local coastal regions in 2001 (substituting for 2000), 2006 (substituting for 2005), 2010, and 2015 were from the National Environmental Bulletins of the Ministry of Environmental Protection of China due to data availability (Table S2). The pollutant discharges include direct discharge of industrial pollution sources, life pollution sources, and comprehensive sewage outlets. City-based economic data, including gross domestic product (GDP) and its components (agricultural, industrial, and service GDPs), and GDP per capita, were collected from the National and Provincial Statistical Yearbooks of China from 1990 to 2015 (Table S2). Pollutant discharges from the coastal regions and main inbound river watersheds and economic data used in this study were listed in Table S4.

### Data processing and analysis

#### Definitions of land conversion and offshore marine surface water quality deterioration of the natural coastal wetland

Land conversion loss of the natural coastal wetland in the three city cluster regions were interpreted from the LandSat images as agricultural and urban lands within boundaries of natural coastal wetland in 1990 when the mainland of China began economic development. The conversional areas were calculated between two neighbor years of investigation, respectively, as new agricultural land use directly from the natural coastal wetland and new urban lands from natural coastal wetland directly and previously converted agricultural land use. The accumulated agricultural and urban lands converted in the past period(s) and left after conversion in the investigated period were also recorded as remaining agricultural and urban lands. Therefore, five land areas were calculated, i.e. new agricultural land from the natural coastal wetland, new urban land from the natural coastal wetland, new urban land from the converted agricultural land use, remaining urban land, and remaining agricultural land.

Annual natural coastal wetland loss was calculated by dividing the total wetland loss in area with the interval years between the two investigated years, such as 10 years for 1990–2000 and 5 years for other periods. Annual conversions of new agricultural land use and new urban land use were also calculated.

Offshore marine surface water quality of coastal wetlands in the three city cluster regions were classified by the National Standards for Marine Water Environmental Quality of China (GB3097-1997, selectively in Table S5). The national standard divides marine water quality into 4 classes. Marine water quality lower than Class III is defined as not usable for industrial and water recreation purposes. Only water quality above Class II is safe for personal dermal contact and aquaculture, etc. Therefore, in this study, the coastal wetlands within the seaward boundary (<6-meter depth below the sea level) in the three city cluster regions were considered as ecologically degraded or deteriorating when their surface water quality was worse than the Class III national standard (GB3097-1997).

### Statistical analysis

Effects of time and location on accumulated land conversion and ecological degradation of the natural coastal wetlands in the three city cluster regions were explored by analysis of variance (ANOVA)-Repeated Measures with variance components as the within-subject covariance structure. Correlations among annual conversional loss of the natural coastal wetland, annual new agricultural land, annual new urban land, and annual economic indices (GDP and its components, and GDP per capita) within the four investigated periods (1990–2000, 2000–2005, 2005–2010, and 2010–2015) were explored using a Pearson correlation analysis. A stepwise regression analysis was used to explore contributions of pollutant discharges (COD, NH_4_^+^-N, TP, and oils) from the local coastal regions (local discharges) and main inbound river watersheds (watershed discharges) to the ecological degradation of the natural coastal wetlands. For the local discharges, GDP and its components and GDP per capita were used as weighting factors separately for the stepwise regression analysis. The pollutants stayed in the stepwise regression models with alpha (partial P value) less than 0.5 when the model P value is less than 0.05. All statistical analyses were done in the R version 3.3.2 (The R Foundation for Statistical Computing).

## Electronic supplementary material

Supplementary Information
