# Supplementary material for: Losses of natural coastal wetlands by land conversion and ecological degradation in the urbanizing Chinese coast
Source: Sci Rep. 2018 Oct 9;8:15046. doi: 10.1038/s41598-018-33406-x (PMC6177474; doi:10.1038/s41598-018-33406-x)
Supplement: Supplementary file 1 — Supplementary Information [file 41598_2018_33406_MOESM1_ESM.docx]

**Losses of natural coastal wetlands by land conversion and ecological degradation in the urbanizing Chinese coast**

Qiaoying Lin, Shen Yu

| **Supplementary Table S1. Basic information of the investigated three coastal city clusters in China and data from the National Bureau of Statistics of China (NBS) in 2015.** | | | | |
| --- | --- | --- | --- | --- |
| City cluster | Coastal province | Number of coastal cities | Percent of the national population | Percent of the national GDP |
| Bohai Rim (BHR) | Tianjin, Liaoning, Hebei, Shandong | 17 | 16.9 | 20.1 |
| Yangtze River Delta (YRD) | Shanghai, Jiangsu, Zhejiang | 9 | 11.6 | 20.1 |
| Pearl River Delta (PRD) | Guangdong | 9 | 7.9 | 10.6 |
| Total | 8 | 35 | 36.4 | 50.8 |

| **Supplementary Table S2. Data sources and usages.** | | | |
| --- | --- | --- | --- |
| Data | Source | Data format | Usage |
| LandSat® images | Global Land Cover Facility (GLCF)  <http://www.landcover.org/data/>  Geospatial Data Cloud site, Computer Network Information Center, Chinese Academy of Sciences  <http://www.gscloud.cn/> | Raster map | To interpret land use and land cover of the coastal wetland regions |
| Global terrain model (ETOPO 1) | NOAA National Geophysical Data Center (NGDC) of USA  <http://www.ngdc.noaa.gov/mgg/global/global.html> | Raster map | To extract the 6-meter depth contour of coastal marine water along the Chinese coastline |
| Google Earth® images | Google Earth®  <http://earth.google.com/> | Raster map | To act as a reference for interpretation and validation processes of land use and land cover in the coastal wetland regions |
| 1:1,000,000 Administrative map of China | National Geometrics Center of China  <http://www.webmap.cn/commres.do?method=dataDownload> | Vector map (polygon) | To define the boundaries of the developed three city clusters, and to register the maps of water quality of offshore marine in China |
| Offshore marine water quality in China | National Environmental Bulletins of China by the Ministry of Environmental Protection of China in 2000, 2005, 2010, and 2015  <http://www.zhb.gov.cn/hjzl/zghjzkgb/lnzghjzkgb/> | Raster map | To obtain offshore marine surface water quality in the residual coastal natural wetlands of the three city cluster regions |
| Pollutant discharges from main inbound rivers to coastal marine | National Marine Environmental Bulletins of China by the State Ocean Administration of China in 2003, 2005, 2010, and 2015  <http://www.soa.gov.cn/zwgk/hygb/> | Table or text file | To obtain discharges of major pollutants from the main inbound rivers to offshore marine |
| Pollutant discharges from coastal regions to coastal marine | National Offshore Marine Environmental Quality Bulletin of China by the Ministry of Environmental Protection of China in 2001, 2006, 2010, and 2015  <http://www.zhb.gov.cn/hjzl/shj/jagb/> | Table or text file | To obtain discharges of major pollutants from the investigated 8 coastal provinces to offshore marine |
| Economic statistics data | National and Provincial Statistical Yearbooks of China by the National and Provincial Bureau of Statistics of China from 1990 to 2015  <http://www.stats.gov.cn/tjsj/ndsj/> | Table or text file | To evaluate the effect of GDP and population on coastal wetlands. City-based data were collected in the 8 investigated coastal provinces. |

| **Supplementary Table S3. Classification system of land use and land cover in the studied coastal city clusters of China.** | |
| --- | --- |
| Types | Subtypes |
| Natural wetland | Tidal marsh, shallow water and estuarine water (< 6-meter depth) |
| Agricultural land | Aquaculture, paddy land, and upland land |
| Urban land | Built-up lands, including residential land, industrial land, commercial land, and traffic land (road, highway, and harbors). |

| **Supplementary Table S4. Pollutant discharges from the coastal regions and trans-provincial river watersheds and economic data in the investigated coastal provinces.** | | | | | | | | | | | | | | |
| --- | --- | --- | --- | --- | --- | --- | --- | --- | --- | --- | --- | --- | --- | --- |
| City cluster | Year^§^ | Annual pollutant discharges from the coastal region (ton) | | | | Annual pollutant discharges from the trans-provincial river watersheds (ton) | | | | Annual GDP and it components in the investigated coastal provinces (billion yuan RMB) | | | | GDP per capita (yuan RMB) |
|  |  | COD^†^ | Oils | NH_4_^+^-N | Total P | COD | Oils | NH_4_^+^-N | Total P | GDP^‡^ | Agricul-tural GDP | Industrial GDP | Service GDP |  |
| BHR | 2000 | 21,914 | 33 | 766 | na^¶^ | 872,140 | 1,610 | 18,410 | 220 | 1,975 | 267 | 989 | 719 | 9,370 |
|  | 2005 | 109,771 | 657 | 12,358 | 1,164 | 579,349 | na | 75,146 | 580 | 4,033 | 436 | 2,175 | 1,422 | 18,880 |
|  | 2010 | 50,093 | 149 | 6,194 | 816 | 549,032 | 5,849 | 12,492 | 1,587 | 8,725 | 793 | 4,676 | 3,255 | 38,852 |
|  | 2015 | 60,000 | 93 | 7,100 | 844 | 283,097 | 619 | 9,950 | 1,549 | 13,802 | 1,101 | 6,462 | 6,239 | 59,487 |
| YRD | 2000 | 13,483 | 130 | 1,325 | na | 2,479,438 | 49,563 | 1,776,237 | 31,482 | 1,313 | 111 | 660 | 542 | 9,646 |
|  | 2005 | 149,380 | 7,826 | 18,273 | 8,313 | 5,216,446 | 33,586 | 89,582 | 44,127 | 2,747 | 154 | 1,481 | 1,111 | 18,985 |
|  | 2010 | 101,028 | 453 | 10,143 | 891 | 10,783,668 | 52,638 | 405,098 | 214,411 | 5,859 | 111 | 660 | 542 | 37,513 |
|  | 2015 | 91,000 | 388 | 3,900 | 1,177 | 6,658,663 | 35,990 | 131,744 | 122,643 | 13,813 | 593 | 5,975 | 7,245 | 86,708 |
| PRD | 2000 | 8,954 | 29 | 194 | na | 1,154,271 | 13,674 | 437,835 | 14,614 | 966 | 100 | 487 | 379 | 11,181 |
|  | 2005 | 71,715 | 825 | 9,039 | 1,238 | 1,830,000 | 42,400 | 108,000 | 22,800 | 2,237 | 143 | 1,134 | 960 | 24,327 |
|  | 2010 | 33,040 | 350 | 2,722 | 57 | 632,016 | 14,045 | 45,007 | 21,801 | 4,601 | 229 | 2,301 | 2,071 | 44,070 |
|  | 2015 | 11,000 | 103 | 1,200 | 299 | 1,913,316 | 12,699 | 38,379 | 18,823 | 7,281 | 335 | 3,261 | 3,685 | 67,115 |
| ^§^The pollutant discharges from watersheds of the Yellow River, the Yangtze River, and the Pearl River in 2000 were not available and substituted by the data of 2003; and the pollutant discharges from local coastal regions in 2000 and 2005 were substituted by data in2001 and 2006, respectively, due to data availability.  ^†^Chemical oxygen demand (COD), petroleum oils (oils), inorganic nitrogen (NH_4_^+^-N), and total phosphorus (Total P) are recorded pollutants by Chinese administrations.  ^‡^GDP means gross domestic product.  ^¶^na means not available. | | | | | | | | | | | | | | |

| **Supplementary Table S5. Selected items of the National Standards for Marine Surface Water Environmental Quality of China (GB3097-1997).** | | | | |
| --- | --- | --- | --- | --- |
| Item | Class I^¶^ | Class II | Class III | Class IV |
| Chemical oxygen demand (COD_Mn_, mg O_2_ L^-1^) ≤ | 2 | 3 | 4 | 5 |
| Biochemical oxygen demand (BOD_5_, mg O_2_ L^-1^) ≤ | 1 | 3 | 4 | 5 |
| Inorganic nitrogen (mg N L^-1^) ≤ | 0.2 | 0.3 | 0.4 | 0.5 |
| Active phosphate (mg P L^-1^) ≤ | 0.015 | 0.03 | 0.03 | 0.045 |
| Mercury (mg Hg L^-1^) ≤ | 0.00005 | 0.0002 | 0.0002 | 0.0005 |
| Cadmium (mg Cd L^-1^) ≤ | 0.001 | 0.005 | 0.01 | 0.01 |
| Lead (mg Pb L^-1^) ≤ | 0.001 | 0.005 | 0.01 | 0.05 |
| Chromium (VI) (mg Cr_VI_ L^-1^) ≤ | 0.005 | 0.01 | 0.02 | 0.05 |
| Arsenic (mg As L^-1^) ≤ | 0.02 | 0.03 | 0.05 | 0.05 |
| Oils (mg L^-1^) ≤ | 0.05 | 0.05 | 0.3 | 0.5 |
| ^¶^The marine surface water environmental quality is classified into 4 classes that characterize application function and protection objective. Class I is applicable to marine fishery waters, marine natural protection areas, and rare marine organism protection areas. Class II is applicable to aquacultural areas, bathing beaches, seas or recreational areas with direct human body sea water contact and industrial waters having direct connection with human food sources. Class III is applicable to industrial water areas and offshore tourist areas. Class IV is applicable to ocean port water areas and ocean exploitation operating areas. | | | | |
